# Diabetic muscle infarction in a 57 year old male: a case report

**DOI:** 10.1186/1756-0500-5-701

**Published:** 2012-12-27

**Authors:** Ivan V Litvinov, Arnold Radu, Natasha Garfield

**Affiliations:** 1 Division of Dermatology, McGill University Health Centre, 687 Pine Ave. West, H7.87, Montreal, Quebec, H3A1A1, Canada; 2 Department of Diagnostic Radiology, McGill University Health Centre, 687 Pine Ave. West, H7.87, Montreal, Quebec, H3A1A1, Canada; 3 Division of Endocrinology, McGill University Health Centre, 687 Pine Ave. West, H7.87, Montreal, Quebec, H3A1A1, Canada

**Keywords:** Diabetic muscle infarction, Diabetic myonecrosis

## Abstract

**Background:**

Diabetic muscle infarction is a rare complication of diabetes mellitus (DM) and is often misdiagnosed as cellulitis. This complication is usually associated with poor disease prognosis and high mortality with previous studies reporting a risk of 50% recurrence or another macrovascular complication occurring within one year. Thus, there needs to be greater awareness of this complication of diabetes.

**Case presentation:**

In the current work, we present a case report and literature review of DMI occurring in a calf of a 57 year old male. However, unlike the suspected trend, our patient has performed well after this incident and has not sustained another macrovascular event now > 15 month since his original diabetic muscle infarction.

**Conclusion:**

Even though diabetic muscle infarction is an uncommon condition, it is important to consider this diagnosis in a diabetic patient. We hope that our findings and literature review will aid clinicians to better diagnose and manage this condition.

## Background

Diabetic muscle infarction (DMI), also known as diabetic myonecrosis, is a rare complication of diabetes mellitus (DM) and is usually associated with poor disease prognosis and high mortality [1,2]. It is often defined as spontaneous ischemic necrosis of skeletal muscle that is unrelated to atheroembolism or occlusion of major arteries [2,3]. The exact prevalence of this condition is not known. A systematic review of the literature from inception to August 2001 identified at total 47 reports describing 166 episodes of DMI [2]. Usually this condition develops with approximate equal frequency in males and females [1,2]. Established risk factors for acquiring a DMI include long-standing DM, insulin dependent type I DM, poor control of glycemia and presence of microvascular diabetic complications (neuropathy, retinopathy, nephropathy) [1,2,4].

Usually, patients present with an acute onset of painful swelling of the thigh (80%), or less commonly the calf (20%), that then evolves over days or weeks [1,2]. Bilateral involvement may occur in ~1/3 of all patients. Rarely a patient may exhibit an involvement of an upper extremity. Fever may be present in 10% cases [1,2]. History of present illness often reveals no trauma or preceding infection. In these patients, even if a DMI is suspected it is critical to rule out pyomyositis, spontaneous gangrenous myositis, clostridial myonecrosis, necrotizing fasciitis and venous thrombosis. It is of notice that diabetic patients are believed to be at increased risk for many of the above conditions [5,6]. A diagnosis of intramuscular hematoma should be considered in patients taking anticoagulation therapy and a possibility of calciphylaxis should be entertained in patients with underlying renal failure. Finally, even though a tumor of the muscle is unlikely to be mistaken for DMI, infarction of a tumor may have a similar presentation.

As evident from above, DMI is in part a diagnosis of exclusion that can be supported by imaging and muscle biopsy. On MRI, one often sees on T2-weighted sequences high intensity signal in the involved muscle, subcutaneous edema and subfascial fluid [1,7-10]. Loss of the normal fatty intramuscular septa is also a common sign of a DMI [1,7-10]. Use of gadolinium in imaging may help distinguish nonenhancing infarcted muscle from surrounding inflammation or edema [1,7-10]. Other imaging modalities including, computer tomography or arteriography are often non-diagnostic and have limited use in confirming a DMI [2,9,10]. Ultrasonography is often used to rule out a venous thromboembolic event, but is not suitable to distinguish between abscess formation and a DMI. Exploration or core muscle biopsy is often performed to support the diagnosis of a DMI and usually shows muscle necrosis, edema and/or occlusion of arterioles and capillaries by fibrin [2].

Few studies have attempted to investigate possible treatment and secondary prevention options for DMIs [1,11]. Unfortunately, currently no unified consensus exists. One retrospective analysis evaluated three possible therapies, which included bed rest and analgesia vs. antiplatelet agents and/or anti-inflammatory medications with rest and analgesia vs. surgical excision of necrotic tissue [1]. The authors report that time to recovery was the shortest when antiplatelet/non-steroidal anti inflammatory (NSAID) agents were used (5 weeks to recovery), while surgical excision correlated with the longest time to recovery (13 weeks). Rest, analgesia and supportive therapy alone yielded intermediate results (8 weeks to recovery). Overall, based on the reviewed literature, there appears to be a trend towards use of small dose of aspirin (80 mg per day) to prevent recurrence of a DMI or other macrovascular complications. NSAIDs are often avoided in these patients due to high risk of precipitating acute kidney injury.

Finally, the benefits of physiotherapy were examined in one study that suggested that straining the involved leg may prolong recovery [12]. However, regular daily activity did not lead to disease exacerbation [12]. Unfortunately, the prognosis for this rare complication of diabetes remains grim with only few patients surviving longer than a year free of another major macrovascular event such as myocardial infarction or a stroke [13]. Furthermore, many patients develop a recurrence of a DMI, with ~50% of cases occurring on a contralateral side [1,2].

In the current report, we describe a case of DMI in a 57 year old male with long standing poorly controlled diabetes. This case highlights clinical and radiological findings that are important in recognising and managing this condition.

## Case presentation

A 57 year old male with 8-year history of poorly controlled Type 2 DM, hypertension, two previous episodes of transient ischemic attacks and underlying cirrhosis of the liver secondary hepatitis C and alcohol abuse presented to our hospital with a two-day history of “not being able to walk”. He reports that his symptoms started about 2 months ago, when one morning he was awoken with a sudden onset of right calf pain, erythema and swelling. He reported his complaints to a family physician, who unsuccessfully treated him with various courses of antibiotics. His pain gradually progressed, until 1 day prior to admission, when he realized that he was no longer able to weight bear on his affected leg. The patient denied any history of fever, trauma, animal bites or infection. Initial work up revealed moderate leukocytosis (white blood count of 14.7 × 10^9 cells/L), normal electrolytes and unremarkable liver function tests. Interestingly, at the time of presentation this patient had normal creatinine kinase (20 U/L), which suggests that the majority of tissue injury occurred during the preceding days to weeks prior to admission. On presentation, the patient had a blood glucose reading of 28.5 mmol/L, while his glycated hemoglobin was 13.9%. Spot urine albumin/creatinin ratio documented significant microalbuminuria Additional diabetic workup failed to reveal any signs of retinopathy or neuropathy.

**Figure 1:**
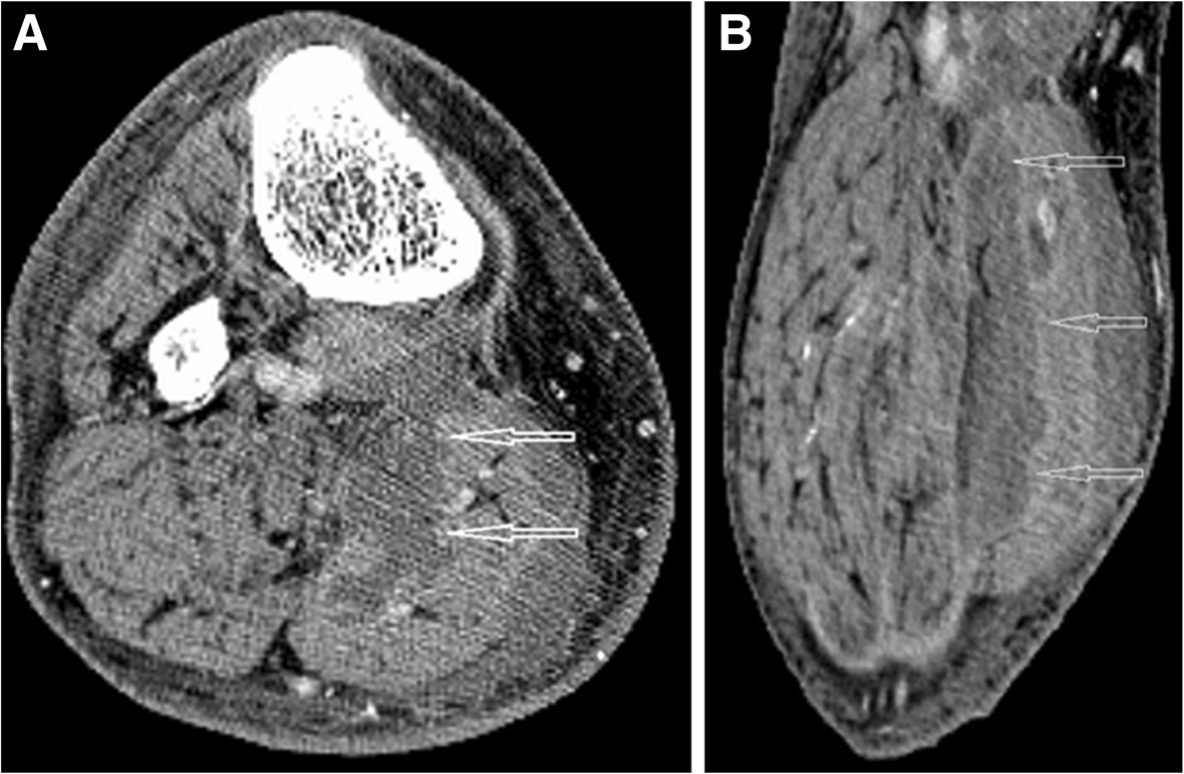
**Selected axial (A) and coronal (B) computer tomography scan images with intravenous contrast demonstrating an ill-defined irregular hypo dense area (arrows) within the medial gastrocnemius muscle extending from the knee to the mid-calf.** The absence of peripheral enhancement and intramuscular gas suggests that this area may represent an abscess.

Blood cultures were obtained and the patient was started empirically on broad spectrum antibiotics. Deep vein thrombosis was ruled out by doppler ultrasound study of both legs. Unfortunately, antibiotic therapy failed to control his symptoms. Subsequently, his cultures showed no bacterial growth and, hence, all antibiotic medications were discontinued. Initial computer tomography imaging of both legs failed to reveal any signs of infection or abscess, but was suggestive of DMI (Figure 1).

**Figure 2:**
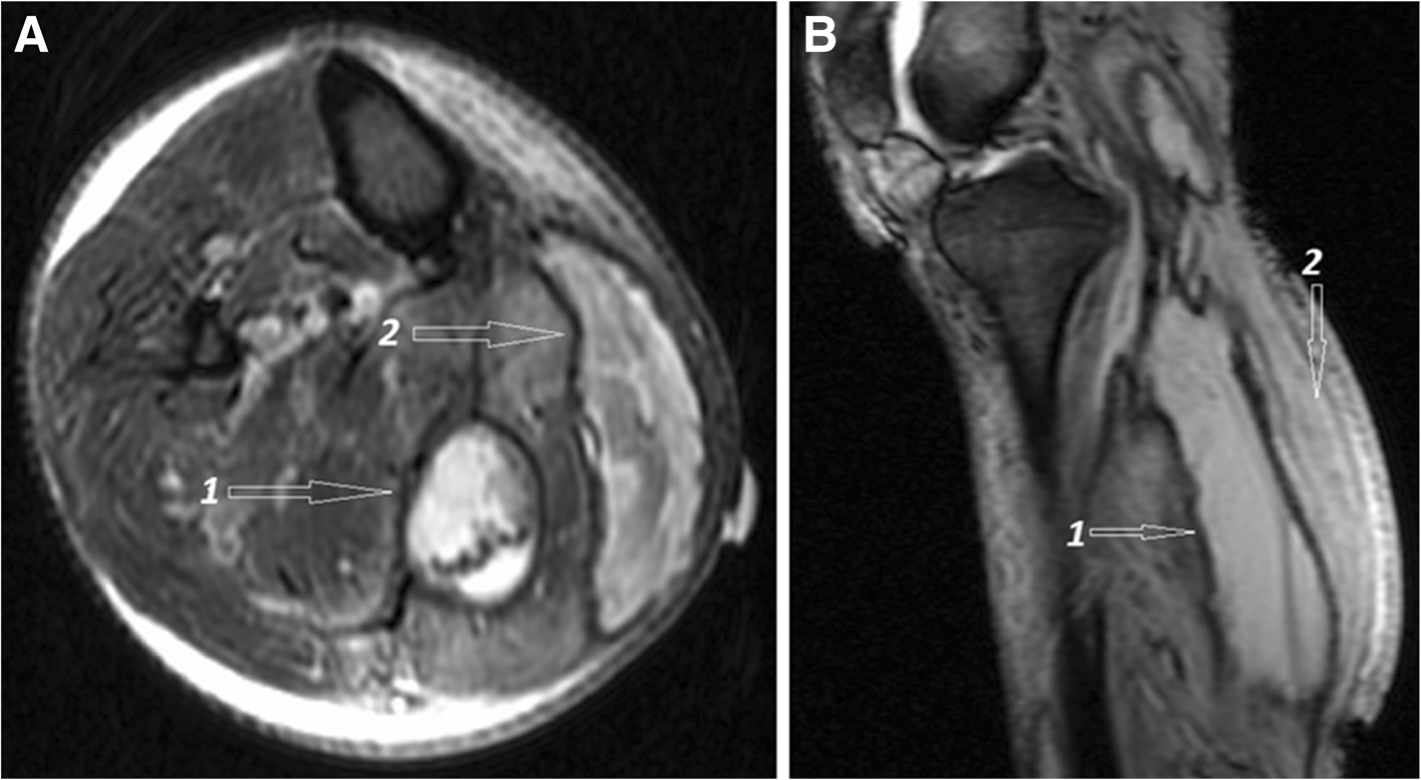
**Selected axial (A) and sagittal (B) magnetic resonance images demonstrating two longitudinal hyper intense collections (1 and 2) within the medial gastrocnemius muscle.** The hyper intense signal of the non-necrotic portions in the gastrocnemius and soleus muscles is indicative of inflammatory changes and edema.

While in hospital, glycemic control was established with intravenous and subcutaneous insulin therapy, pain was controlled with analgesic medication and bed rest. Subsequent MRI revealed significant edema, inflammation, necrosis of gastrocnemious and soleus muscles and identified a number of associated communicating fluid collections, that were deemed to be at high risk of becoming infected (Figure 2). There was no evidence of arterial thromboembolism. Due to the presence of fluid collections that were deemed to be at high risk of becoming infected, plastic surgery service was consulted and wound debridement was performed. Pathological analysis of debris obtained from soleus and gastrocnemius muscles revealed necrotic tissue with signs of severe acute and chronic inflammation (data not shown). Bacterial and fungal stains and cultures for these tissues did not reveal any infectious agents. The patient gradually recovered over the period of ~4 weeks in the hospital with supportive therapy and was discharged to a rehabilitation facility on insulin, clopidogrel and Aspirin (80 mg per day). He had no further complications on his 15 months follow up visit and is followed regularly in our diabetic clinic.

## Conclusion

In summary, we presented a case of DMI in Type 2 diabetic patient, who presented with right calf involvement, but performed significantly better then expected with respect to his diabetes progression. Even though DMI is an uncommon condition, it is important to consider this diagnosis in a diabetic patient. Presence of the aforementioned risk factors and lack of response to antibiotics may play an important role in establishing a diagnosis. We hope that our findings and literature review will aid clinicians to better diagnose and manage this condition.

## Consent

Written informed consent was obtained from the patient for publication of this Case Report and any accompanying images. A copy of the written consent is available for review by the Editor-in-Chief of this journal.

## Abbreviations

DMI: Diabetic muscle infarction; DM: Diabetes mellitus.

## Competing interest

The authors declare no competing financial interests.

## Authors’ contributions

IVL analyzed and interpreted patient data, conducted literature review and prepared the manuscript. AR assisted in selecting and interpreting radiological images related to this case and was a significant contributor in writing the manuscript. NG supervised patient data acquisition and interpretation, provided expert opinion in literature review and manuscript structure and was a major contributor in writing the manuscript. All authors read and approved the final manuscript.
